# Simultaneous Determination of Stilbenes, Flavones, Coumestans, Anthraquinones, and Chalcones in Ethanolic Extract of Pet-Sang-Kard Mixed Herbal Remedy Using HPLC-PDA Analysis

**DOI:** 10.3390/molecules30020222

**Published:** 2025-01-08

**Authors:** Weerasak Samee, Wanna Eiamart, Sarin Tadtong, Chuda Chittasupho

**Affiliations:** 1 Department of Pharmaceutical Chemistry, Faculty of Pharmacy, Srinakharinwirot University, Nakhon Nayok 26120, Thailand; 2 Chula Pharmacokinetic Research Center, Faculty of Medicine, Chulalongkorn University, Bangkok 10330, Thailand; wanna.eiamart@g.swu.ac.th; 3 Department of Pharmacognosy, Faculty of Pharmacy, Srinakharinwirot University, Nakhon Nayok 26120, Thailand; sarin@g.swu.ac.th; 4 Department of Pharmaceutical Sciences, Faculty of Pharmacy, Chiang Mai University, Chiang Mai 50200, Thailand; chuda.c@cmu.ac.th

**Keywords:** stilbenes, flavones, coumestans, anthraquinones, chalcones, chromatography

## Abstract

The Pet-Sang-Kard mixed herbal remedy (PSKMHR) is a traditional Thai medicinal formulation used as an herbal supplement for the treatment of hemorrhoids. This remedy consists of four specific herbal ingredients in the following proportions: 50 parts *Cissus quadrangularis* L. stems, 15 parts *Eclipta prostrata* L. aerial parts, 10 parts *Rheum* sp. rhizome, and 10 parts *Boesenbergia rotunda* (L.) Mansf. rhizome. This study presents the development, validation, and application of a high-performance liquid chromatography with photodiode array detection (HPLC-PDA) method designed for the simultaneous quantification of 13 key bioactive compounds, including rhaponticin, rhapontigenin, quercitrin, wedelolactone, aloe-emodin, rhein, emodin, chrysophanol, physcion, alpinetin, pinocembrin, pinostrobin, and panduratin A, present in the 70% ethanolic extract of PSKMHR. Method validation was conducted in accordance with Association of Official Analytical Collaboration (AOAC) international guidelines, evaluating parameters such as the specificity, linearity, accuracy, precision, and limit of detection. The results demonstrated exceptional linearity (R > 0.9999), high precision (% RSD < 2), and recovery rates within acceptable limits (98–102%) for all analytes. This developed method was successfully applied to quantify the 13 target compounds in the crude extracts of PSKMHR formulated from 10 market raw material samples, providing a robust analytical framework for quality control of this herbal remedy.

## 1. Introduction

**Figure 1:**
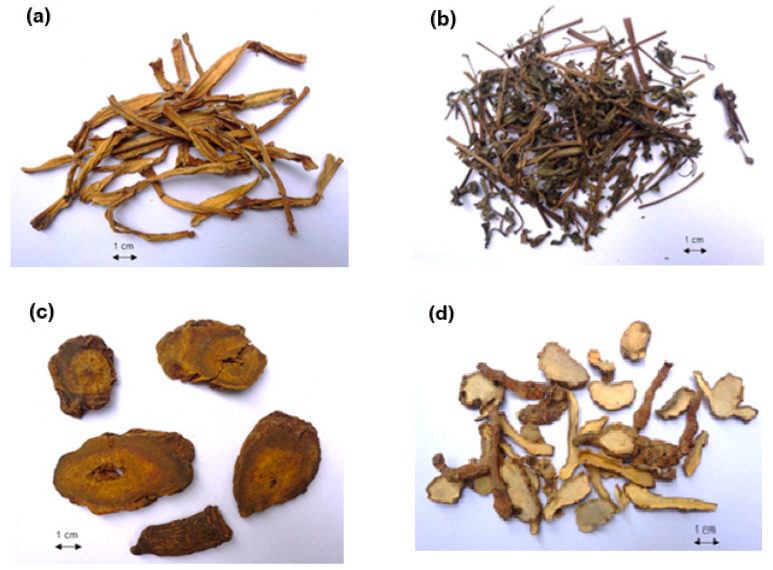
Raw materials of PSKMHR: (**a**) *C. quadrangularis* L. stems, (**b**) *E. prostrata* L. aerial parts, (**c**) *Rheum* sp. rhizome, and (**d**) *B. rotunda* (L.) Mansf. rhizome.

Herbal medicine has been utilized for centuries in the management of hemorrhoids, with numerous plants acknowledged for their therapeutic efficacy. Among these, *Cissus quadrangularis* L. is prominently featured in the Thai National List of Herbal Products due to its demonstrated effectiveness in clinical settings [1]. This aligns with broader findings supporting the use of various botanical remedies for hemorrhoidal disease, which is characterized by pain and inflammation [2]. Traditionally, the application of herbal treatments has involved the use of decoctions and topical preparations [3]. The incorporation of herbal remedies into modern medical practices is expanding, facilitated by research into their phytochemical constituents and biological activities [4]. Specifically, in Thailand, the Pet-Sang-Kard mixed herbal remedy (PSKMHR), a formulation encompassing four herbs—50 parts *Cissus quadrangularis* L. stems, 15 parts *Eclipta prostrata* L. aerial parts, 10 parts *Rheum* sp. rhizome, and 10 parts *Boesenbergia rotunda* (L.) Mansf. rhizome—is officially recognized for the treatment of hemorrhoids, underscoring the integration of traditional and contemporary therapeutic approaches (a picture of raw materials is shown in Figure 1) [5].

*C. quadrangularis*, a plant belonging to the Vitaceae family, is recognized for its anti-inflammatory and antioxidant properties. Its principal component, quercitrin (quercetin-3-*O*-rhamnoside), contributes to these health benefits [6]. The plant’s anti-inflammatory effects function through various mechanisms, such as the following: 1. Modulating inflammatory pathways by suppressing NF-κB activation; 2. Inhibiting cyclooxygenase (COX) and lipoxygenase (5-LOX) enzymes; 3. Reducing edema in animal models; and 4. Scavenging free radicals and reducing oxidative stress [6,7,8,9,10]. Clinical studies have demonstrated its efficacy in treating hemorrhoids, attributed to its anti-inflammatory, analgesic, and vasoconstrictive properties [1]. The plant’s historical use in traditional medicine and its potential applications in modern healthcare underscore its versatility as a medicinal agent for managing inflammatory conditions [11]. *E. prostrata*, an Asteraceae family herb, has proven anti-inflammatory properties. Its key component, wedelolactone, inhibits inflammatory mediators by suppressing the NF-κB pathway [12,13]. The herb protects bronchial cells from smoke-induced inflammation and contains coumestan compounds that regulate inflammatory responses [14]. The herb’s antioxidant properties also contribute to its anti-inflammatory effects [15]. Overall, *E. prostrata* shows promise in treating various inflammatory conditions through multiple mechanisms, making it valuable in both traditional and modern medicine. Rhubarb (*Rheum* sp.) is a medicinal herb known for its anti-inflammatory properties, primarily due to anthraquinones like emodin and rhein. It inhibits pro-inflammatory cytokines such as TGF-β1, CTGF, IL-6, and TNF-α [16,17]. The herb modulates inflammatory pathways, aiding in conditions like chronic renal failure and wound healing. Rhubarb phenol 8-*O*-glucoside inhibits STAT3 signaling, reducing liver inflammation and fibrosis [18]. Rhein, another compound in *Rheum officinale*, shows anti-inflammatory, antioxidative, and anti-tumor effects [19]. Additionally, rhubarb contains stilbene compounds such as rhaponticin, resveratrol, and rhapontigenin, with rhaponticin and rhapontigenin being the two most abundant stilbenes found in rhubarb [20]. Rhaponticin, a glycoside, is metabolized into rhapontigenin, which is considered the biologically active form. Research indicates that rhapontigenin exhibits significant antioxidant activity by scavenging reactive oxygen species (ROS), thereby protecting cells from oxidative stress [21,22]. This antioxidant capability is crucial in mitigating various diseases linked to oxidative damage, including cancer and cardiovascular diseases [23,24]. In addition to its antioxidant effects, rhapontigenin demonstrates anti-inflammatory properties. It has been reported to inhibit the MAPK/NF-κB signaling pathways in human endothelial cells, which are crucial in the inflammatory response [23]. This inhibition can help reduce inflammation associated with chronic diseases, including diabetes and cardiovascular disorders [24]. Moreover, rhaponticin has been linked to protective effects against retinal oxidative stress and inflammation in diabetic models, further emphasizing its therapeutic potential [24]. Overall, rhubarb’s ability to modulate inflammatory responses through multiple mechanisms makes it valuable in both traditional and modern medicine for treating various inflammatory conditions. Finger root (*B. rotunda*) has potent anti-inflammatory properties due to its bioactive compounds, especially flavonoids and chalcones like pinostrobin and panduratin A. It inhibits inflammatory enzymes such as cyclooxygenase-2 (COX-2) and 5-lipoxygenase and reduces nitric oxide synthase production [25,26,27,28,29]. The plant’s antioxidant properties also contribute to its anti-inflammatory effects by scavenging free radicals and reducing oxidative stress [30,31,32]. In vivo studies have shown *B. rotunda*’s ability to alleviate inflammation in animal models by reducing inflammatory markers like Akt and NF-kappaB p65 [33]. Its multifaceted action in reducing inflammation and oxidative stress makes it a promising candidate for treating various inflammatory conditions and protecting against cardiotoxicity [30].

The simultaneous quantification of compounds using high-performance liquid chromatography with photodiode array detection (HPLC-PDA) is a powerful analytical technique that offers several advantages in the analysis of complex mixtures, particularly in the fields of pharmaceuticals, food science, and natural product research. Its key advantages include the following: 1. High sensitivity and specificity for detecting multiple analytes. 2. Ability to simultaneously analyze various compounds in a single run. 3. Excellent reproducibility and reliability, crucial for quality control. 4. Robust quantitative capabilities with high accuracy. 5. Versatility in analyzing a wide range of compounds and sample types. 6. Cost-effectiveness compared to other advanced analytical techniques. This method enhances efficiency, accuracy, and reliability in chemical analyses across various fields, making it essential for research and quality control laboratories [34,35,36,37].

**Figure 2:**
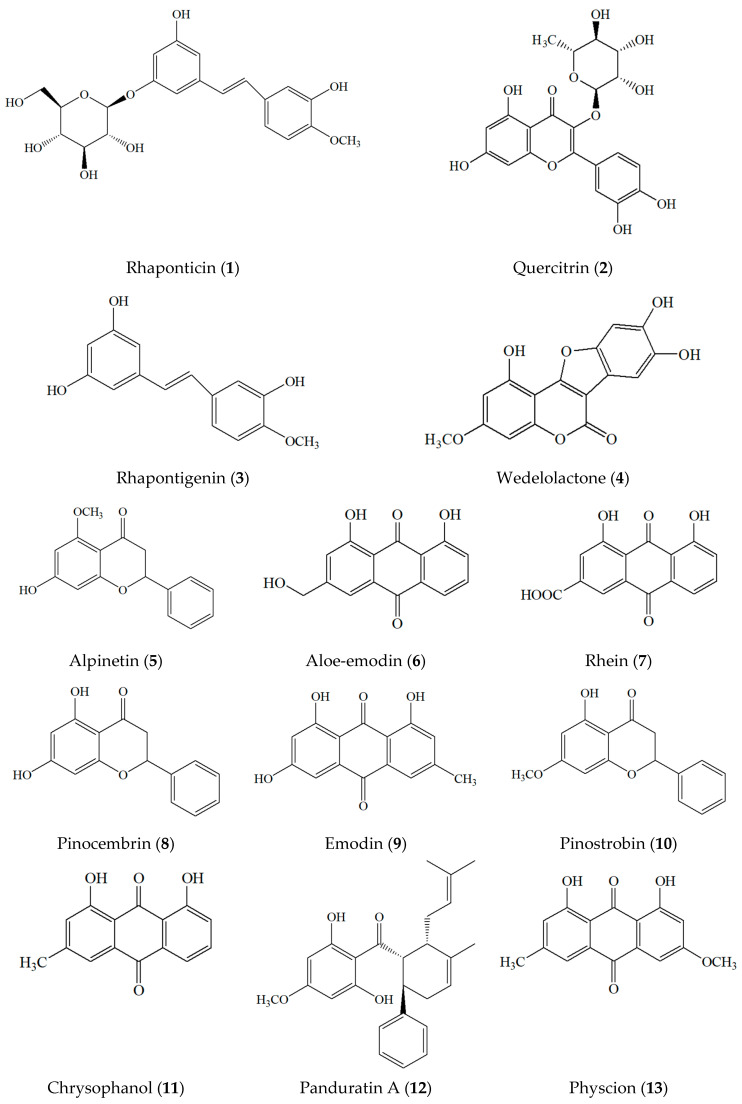
Chemical structures of rhaponticin (**1**), quercitrin (**2**), rhapontigenin (**3**), wedelolactone (**4**), alpinetin (**5**), aloe-emodin (**6**), rhein (**7**), pinocembrin (**8**), emodin (**9**), pinostrobin (**10**), chrysophanol (**11**), panduratin A (**12**), and physcion (**13**).

The four herbs present in PSKMHR exhibit significant antioxidant and anti-inflammatory properties. This study involved the preparation of an ethanolic extract from a mixture of four herbs purchased from ten herbal shops in Thailand, as well as the analysis of thirteen major compounds identified in PSKMHR. PSKMHR encompasses a diverse range of compounds characterized by varying polarities and UV absorbance properties. Therefore, we utilized the HPLC-PDA technique to develop a method for the simultaneous analysis of all four types of herbal mixtures. The simultaneous detection of rhaponticin (**1**), quercitrin (**2**), rhapontigenin (**3**), wedelolactone (**4**), alpinetin (**5**), aloe-emodin (**6**), rhein (**7**), pinocembrin (**8**), emodin (**9**), pinostrobin (**10**), chrysophanol (**11**), panduratin A (**12**), and physcion (**13**) using HPLC with a photodiode array detector has not been previously reported (the chemical structures of the analytes are shown in Figure 2). Therefore, the objective of this study was to develop an HPLC-PDA method for the simultaneous detection of these 13 major compounds for quality control of PSKMHR preparations. Validation was conducted in accordance with Association of Official Analytical Collaboration (AOAC) international guidelines [38].

## 2. Results and Discussion

### 2.1. Selection of Marker Analytes for Quality Control of PSKMHR Using HPLC-PDA System

**Figure 3:**
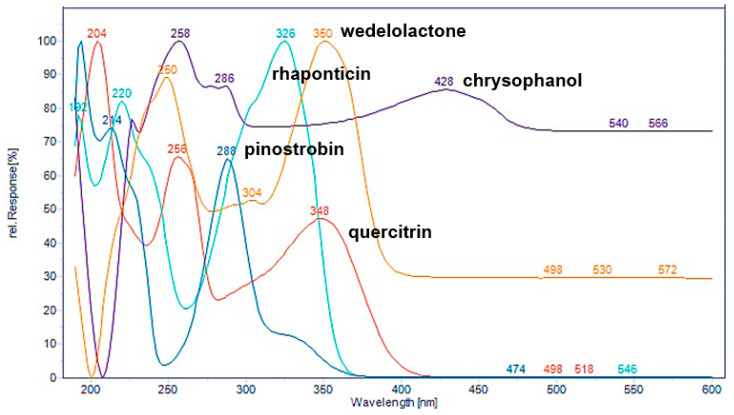
UV spectrum of representative compounds of stilbene (rhaponticin), flavone (quercitrin), coumestan (wedelolactone), chalcone (pinostrobin), and antraquinone (chrysophanol).

**Figure 4:**
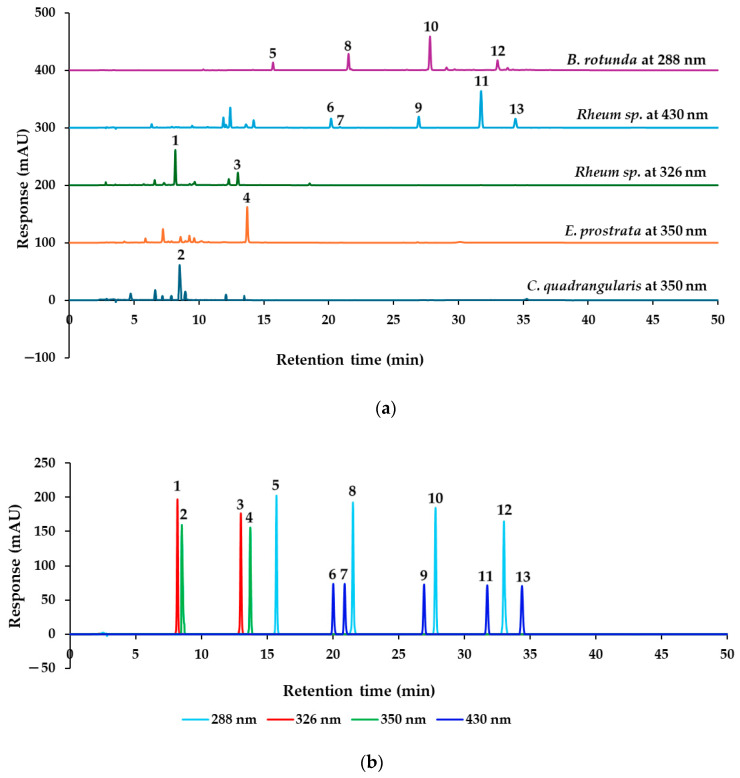
Typical HPLC chromatograms of (**a**) *C. quadrangularis* at 350 nm, *E. prostrata* at 350 nm, *Rhubarb* sp. at 326 nm, *Rhubarb* sp. at 430 nm, and *B. rotunda* at 288 nm. (**b**) Overlay chromatogram of four groups of reference standards categorized by maximum absorption wavelengths: rhaponticin (**1**) at 326 nm, quercitrin (**2**) at 350 nm, rhapontigenin (**3**) at 326 nm, wedelolactone (**4**) at 350 nm, alpinetin (**5**) at 288 nm, aloe-emodin (**6**) at 430 nm, rhein (**7**) at 430 nm, pinocembrin (**8**) at 288 nm, emodin (**9**) at 430 nm, pinostrobin (**10**) at 288 nm, chrysophanol (**11**) at 430 nm, panduratin A at 288 nm (**12**), and physcion (**13**) at 430 nm.

The marker analytes in each herb for the HPLC analysis were selected from the literature and represent specific compounds in each herb as follows: quercitrin from *C. quadrangularis* [6], wedelolactone from *E. prostrata* [12], two stilbenes (rhaponticin and rhapontigenin), and five anthraquinones (aloe-emodin, rhein, emodin, chrysophanol, and physcion) from *Rheum* sp. [20], in addition to alpinetin, pinocembrin, pinostrobin, and panduratin A from *B. rotunda* [25]. To confirm the presence of these marker analytes in each type of herbal medicine, we analyzed the 70% ethanolic extracts of the herbs using HPLC-PDA. Based on the maximum absorption wavelengths of the reference standards (Figure 3), the thirteen reference standards were categorized into four groups according to their absorption wavelength results. The analysis was conducted at UV wavelengths of 350 nm for quercitrin and wedelolactone, 288 nm for pinostrobin and other chalcone derivatives, 326 nm for rhaponticin and the stilbene rhapontigenin, and 430 nm for chrysophanol and other anthraquinones. Each group of reference standards was separated for the analysis to confirm the presence of these compounds in herbal samples. This analysis utilized a reversed-phase C18 column and gradient elution with a mobile phase consisting of ultrapure water and acetonitrile (both containing 0.1% formic acid) at a flow rate of 1 mL/min. The presence of the target components in each constituent herbal medicine was confirmed (Figure 4a). These findings were then compared with the overlay chromatograms of the four groups of reference standards, as illustrated in Figure 4b.

### 2.2. Optimization of UV Detection for Analysis of Marker Analytes in PSKMHR

#### 2.2.1. Effect of Individual Herbal Components on Chromatographic Fingerprint of PSKMHR at 288 nm UV Detection

**Figure 5:**
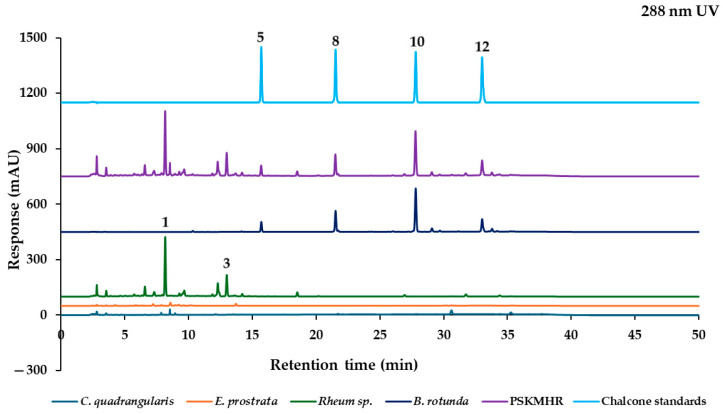
Chromatograms of *C. quadrangularis*, *E. prostrata*, *Rhubarb* sp., *B. rotunda*, PSKMHR, and chalcone standards at 288 nm.

The chalcone compounds exhibit a maximum absorbance at approximately 288 nm. As illustrated in Figure 5, these chalcone compounds were identified in both *B. rotunda* and PSKMHR. Furthermore, the compounds rhaponticin (**1**) and rhapontigenin (**3**) were also detected in PSKMHR. In conclusion, the chromatogram of PSKMHR clearly highlights the presence of chalcone compounds, including alpinetin (**5**), pinocembrin (**8**), pinostrobin (**10**), and panduratin A (**12**) from the rhizome of *B. rotunda*. Additionally, stilbene compounds such as rhaponticin (**1**) and rhapontigenin (**3**) sourced from the rhizome of *Rheum* sp. were observed.

#### 2.2.2. Effect of Individual Herbal Components on Chromatographic Fingerprint of PSKMHR at 326 nm UV Detection

**Figure 6:**
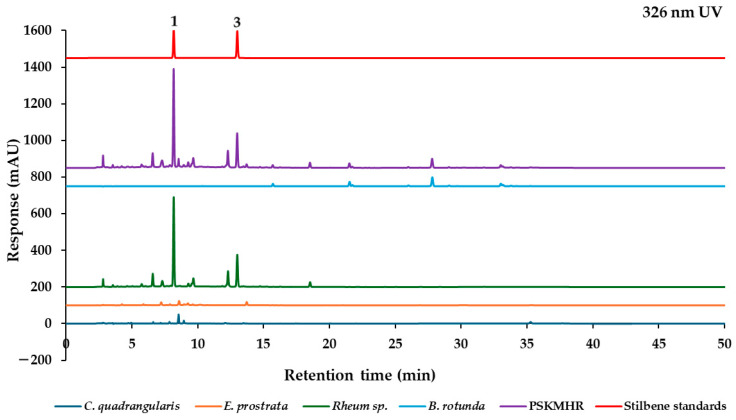
Chromatograms of *C. quadrangularis*, *E. prostrata*, *Rhubarb* sp., *B. rotunda*, PSKMHR, and stilbene standards at 326 nm.

The stilbene compounds demonstrate a maximum absorbance at approximately 326 nm. Figure 6 illustrates the identification of these stilbene compounds in both *Rheum* sp. and PSKMHR. The chromatograms for PSKMHR and the rhizome of *Rheum* sp. at 326 nm detection clearly indicate the presence of stilbene compounds, including rhaponticin (**1**) and rhapontigenin (**3**).

#### 2.2.3. Effect of Individual Herbal Components on Chromatographic Fingerprint of PSKMHR at 430 nm UV Detection

**Figure 7:**
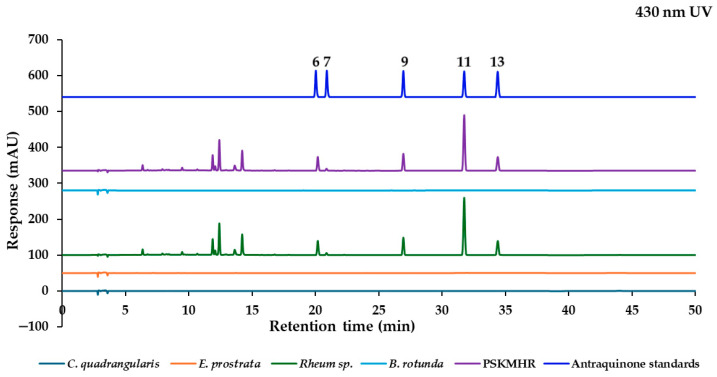
Chromatograms of *C. quadrangularis*, *E. prostrata*, *Rhubarb* sp., *B. rotunda*, PSKMHR, and anthraquinone standards at 430 nm.

The anthraquinones exhibit a maximum absorbance at approximately 430 nm. Figure 7 illustrates the identification of these anthraquinones in both *Rheum* sp. and PSKMHR. The chromatograms for PSKMHR and the rhizome of *Rheum* sp. at 430 nm detection distinctly indicate the presence of anthraquinones, including aloe-emodin (**6**), rhein (**7**), emodin (**9**), chrysophanol (**11**), and physcion (**13**).

#### 2.2.4. Effect of Individual Herbal Components on Chromatographic Fingerprint of PSKMHR at 350 nm UV Detection

**Figure 8:**
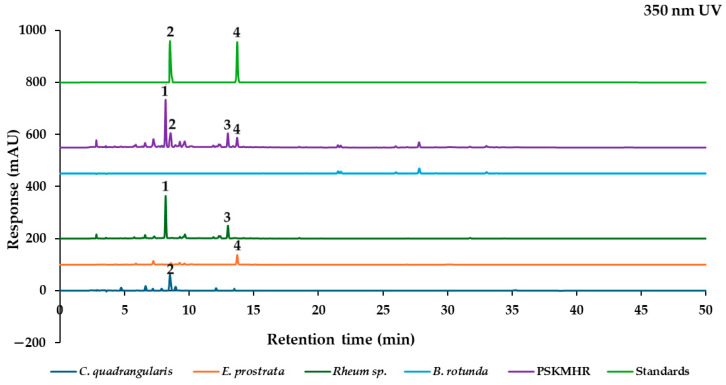
Chromatograms of *C. quadrangularis*, *E. prostrata*, *Rhubarb* sp., *B. rotunda*, PSKMHR, and standards (quercitrin (**2**) and wedelolactone (**4**)) at 350 nm.

Quercitrin (**2**) and wedelolactone (**4**) exhibit a maximum absorbance at approximately 350 nm. As illustrated in Figure 8, quercitrin was identified in both *C. quadrangularis* and PSKMHR, while wedelolactone was detected in both *E. prostrata* and PSKMHR. Additionally, the compounds rhaponticin (**1**) and rhapontigenin (**3**) were also identified in PSKMHR. In conclusion, the chromatogram of PSKMHR distinctly highlights the presence of quercitrin (**2**) derived from *C. quadrangularis*, wedelolactone (**4**) sourced from *E. prostrata*, and stilbene compounds such as rhaponticin (**1**) and rhapontigenin (**3**) from the rhizome of *Rheum* sp.

Based on the findings presented in Figure 3, Figure 4, Figure 5, Figure 6, Figure 7 and Figure 8, it is concluded that specific wavelengths should be utilized for the quantitative analysis of the 13 key compounds in PSKMHR via an HPLC-PDA analysis. The optimal wavelengths identified are as follows: 288 nm for the analysis of alpinetin (**5**), pinocembrin (**8**), pinostrobin (**10**), and panduratin A (**12**); 326 nm for the analysis of rhaponticin (**1**) and rhapontigenin (**3**); 430 nm for aloe-emodin (**6**), rhein (**7**), emodin (**9**), chrysophanol (**11**), and physcion (**13**); and 350 nm for quercitrin (**2**) and wedelolactone (**4**).

### 2.3. Method Validation of Developed HPLC Analytical Method

The differing polarities of stilbenes, flavones, coumestans, chalcones, and anthraquinones were successfully separated using a C18 HPLC column (250 × 4.6 mm, 5.0 µm). The mobile phase consisted of 0.1% formic acid in ultrapure water (phase A) and 0.1% formic acid in acetonitrile (phase B). The pretreatment sample was analyzed under the following gradient conditions: a gradient from 20% to 80% phase B over 30 min, maintained at 80% phase B for an additional 5 min, followed by a gradient returning to 20% phase B over 5 min, and finally returning to 20% phase B for 10 min. The analysis was performed at a flow rate of 1 mL/min, within a detection wavelength range of 200–600 nm (with representative wavelengths at 288, 326, 350, and 430 nm) and at a column temperature maintained at 25 °C, resulting in a resolution greater than 2.

In the standard chromatographic analysis of reference standards conducted at 288 nm, as illustrated in Figure 4, the retention times for alpinetin, pinocembrin, pinostrobin, and panduratin A were recorded as follows: 15.69 min, 21.51 min, 27.79 min, and 33.00 min, respectively. When analyzed at 326 nm, two peaks corresponding to rhaponticin and rhapontigenin were observed at retention times of 8.17 min and 12.99 min, respectively. At 350 nm, quercitrin and wedelolactone exhibited retention times of 8.56 min and 13.71 min, respectively. Finally, at 430 nm, five peaks were identified as aloe-emodin, rhein, emodin, chrysophanol, and physcion, appearing at retention times of 20.17 min, 20.86 min, 26.93 min, 31.73 min, and 34.37 min, respectively.

The HPLC-PDA method was validated in accordance with AOAC guidelines with respect to linearity, limits of detection (LODs), limits of quantification (LOQs), precision, and accuracy.

Linearity was evaluated using five-point calibration curves with concentration levels determined based on the expected amounts of analytes in the PSKMHR extracts derived from preliminary studies. As shown in Table 1, the coefficient of determination (R^2^), which assesses linearity, demonstrated excellent linearity, ranging from 0.9999 to 1.0000 for all markers based on the prepared calibration curve.

The LODs and LOQs for the thirteen marker components investigated were found to be between 0.0032 and 0.0134 µg/mL and 0.0100 and 0.0405 µg/mL, respectively (Table 1). The selection of specific wavelengths for the analysis of each compound group enhanced the specificity and sensitivity of the analysis, thereby maximizing the benefits of the PDA detector.

The recovery percentages for compounds 1 to 13 ranged from 98.79% to 102.00%, as summarized in Table 2. The validation of precision was evaluated using the relative standard deviation (RSD). All RSD values associated with repeatability and both intra-day and inter-day precision for the investigated markers were found to be within 2.00%, indicating satisfactory precision validation results (Table 2). This validation data confirms that the developed HPLC assay is an appropriate and accurate method for quantifying the selected marker substances in the quality control of PSKMHR market samples.

### 2.4. Analysis of PSKMHR from Different Sources of Raw Materials

**Figure 9:**
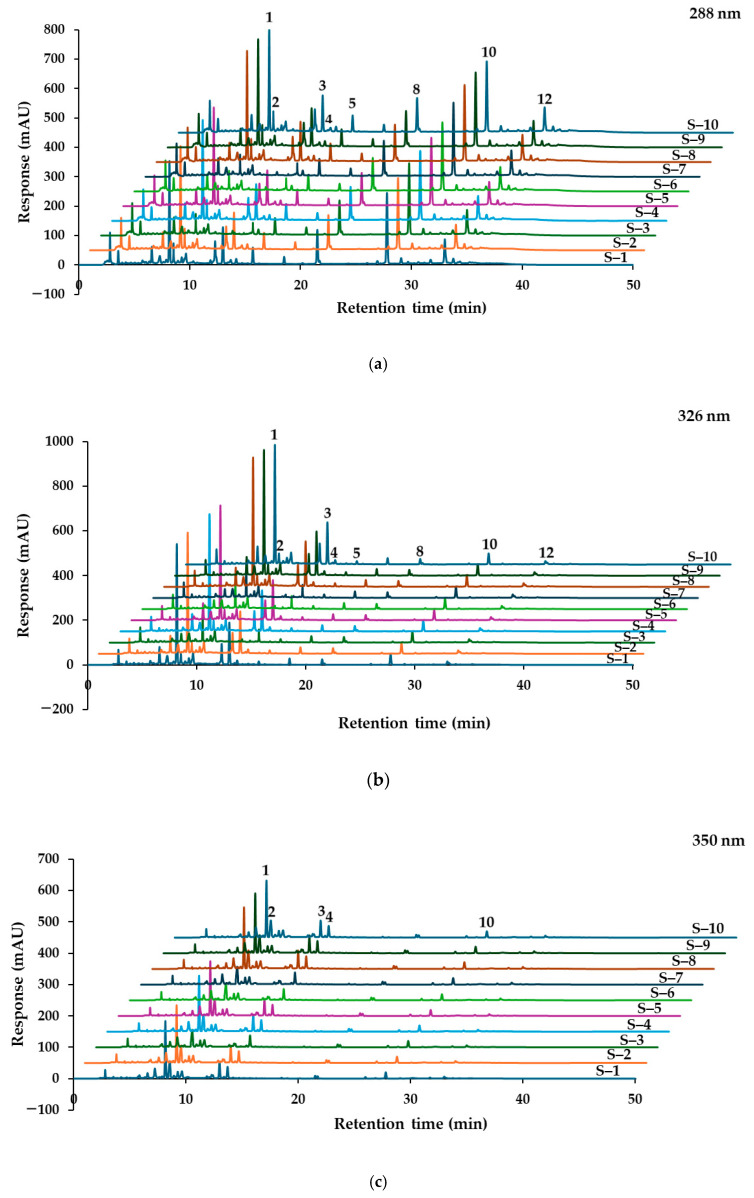

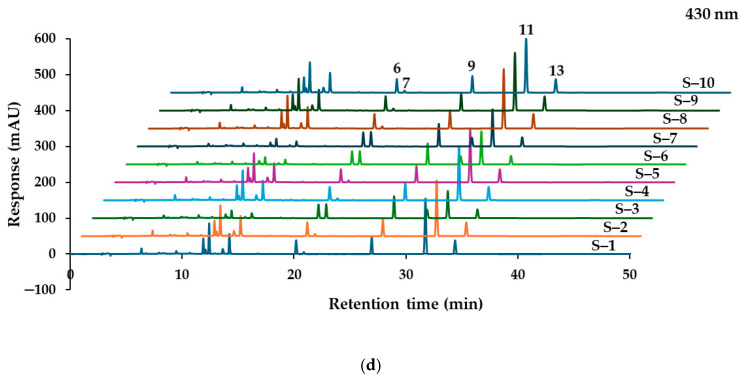
HPLC chromatograms of PSKMHR formulated from ten market raw material samples, detected at (**a**) 288 nm, (**b**) 326 nm, (**c**) 350 nm, and (**d**) 430 nm.

Ten PSKMHR samples, labeled S–1 to S–10, were prepared from various sources of raw materials and extracted using ultrasonic-assisted extraction with 70% ethanol in water. These pretreated samples were analyzed using a validated HPLC-PDA method at a concentration of 10 mg/mL based on dry weight. The chromatograms are presented in Figure 9a–d, and the quantitative results for the thirteen markers are detailed in Table 3.

At a UV detection wavelength of 288 nm, the concentrations of alpinetin (**5**) ranged from 156.63 to 176.53 µg/g of dry weight, pinocembrin (**8**) ranged from 372.82 to 419.92 µg/g, pinostrobin (**10**) ranged from 845.94 to 953.00 µg/g, and panduratin A (**12**) ranged from 272.69 to 307.15 µg/g of dry weight. At a UV detection wavelength of 326 nm, rhaponticin (**1**) was detected at levels ranging from below the limit of quantification (LOQ) to 952.72 µg/g of dry weight, while rhapontigenin (**3**) ranged from below the LOQ to 322.49 µg/g of dry weight.

At a UV detection wavelength of 350 nm, quercitrin (**2**) concentrations varied from 169.33 to 203.11 µg/g of dry weight, and wedelolactone (**4**) ranged from 238.81 to 269.06 µg/g of dry weight. At a UV detection wavelength of 430 nm, the amounts of aloe-emodin (**6**) ranged from 19.71 to 22.24 µg/g of dry weight, rhein (**7**) ranged from 2.71 to 21.99 µg/g, emodin (**9**) ranged from 26.87 to 44.12 µg/g, chrysophanol (**11**) ranged from 50.44 to 100.62 µg/g, and physcion (**13**) ranged from 17.42 to 30.23 µg/g of dry weight.

Based on the chromatograms presented in Figure 9 and the quantified amounts of the thirteen markers listed in Table 3, the PSKMHR samples were categorized into two groups. Samples S–3, S–6, and S–7 were classified as Group 1, whereas samples S–1, S–2, S–4, S–5, S–8, S–9, and S–10 were classified as Group 2. In Group 1, the peaks corresponding to rhaponticin (**1**) and rhapontigenin (**3**) were detected below the limit of quantification (LOQ), while rhein (7) was present at concentrations approximately seven times higher than those found in Group 2, and emodin (9) was approximately 1.5 times more concentrated. Conversely, the levels of chrysophanol (11) and physcion (13) in Group 1 were about 1.5 times lower than those in Group 2.

This classification may be attributed to the use of different species of rhubarb samples. As reported in the study by Au et al. (2024), official rhubarb varieties (*R. palmatum*, *R. tanguticum*, and *R. officinale*) contain five anthraquinones, including aloe-emodin, emodin, rhein, chrysophanol, and physcion. In contrast, unofficial rhubarb varieties (such as *R. rhaponticum*, *R. rhabarbarum*, *R. emodi*, etc.) exhibit elevated levels of rhapontigenin and anthraquinones, particularly chrysophanol, with no detectable levels of rhein [20].

**Figure 10:**
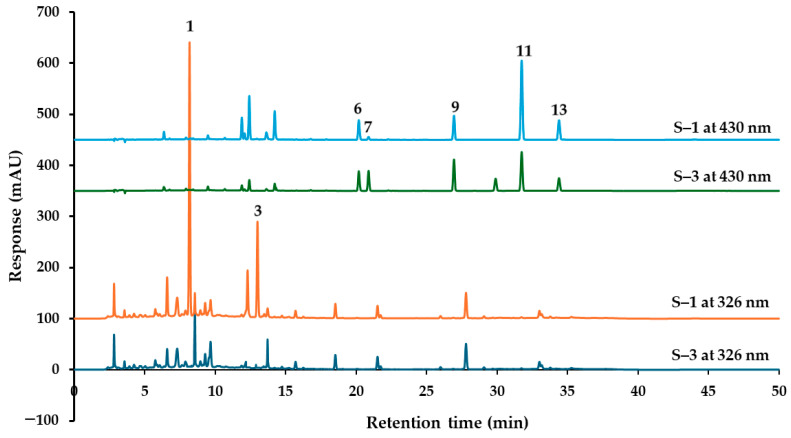
HPLC chromatograms of PSKMHR samples S–1 and S–3 detected at 326 nm, and 430 nm.

To compare the differences between the two groups, sample S–1 represents Group 1 and sample S-3 represents Group 2. The chromatogram illustrated in Figure 10 shows that at 326 nm, S–1 exhibits major peaks for rhaponticin (**1**) and rhapontigenin (**3**), while these peaks are undetectable in S–3. Furthermore, at 430 nm, S–1 presents a smaller peak for rhein compared to that in S–3.

These findings enable differentiation between various sources of rhubarb. Consequently, it is essential for manufacturers to verify the concentration of active compounds prior to the formulation of PSKMHR herbal remedies or other medications that include rhubarb as an ingredient. This step is necessary to ensure the pharmacological efficacy intended from the specific chemical components present in each rhubarb species.

## 3. Materials and Methods

### 3.1. Reagents

Acetonitrile of chromatography grade was purchased from Merck (Darmstadt, Germany). Formic acid, rhaponticin, rhapontigenin, rhein, emodin, and panduratin A were obtained from Sigma-Aldrich^®^ Co. (St. Louis, MO, USA). Quercitrin, wedelolactone, alpinetin, aloe-emodin, pinocembrin, pinostrobin, chrysophanol, and physcion were acquired from ChemFaces^®^ (Wuhan, China).

### 3.2. Preparation of PSKMHR

A total of ten sources of raw materials were prepared for the ten formulations of PSKMHR, designated as S1 to S10. Each raw material was processed in a cutting mill and sieved through a 60-mesh filter to obtain a fine powder, which was then stored in a tightly sealed container for future use. The preparation of PSKMHR involved weighing a blend of herbal powders consisting of four specific ingredients in predetermined proportions: 50 parts *C. quadrangularis* L. stems, 15 parts *E. prostrata* L. aerial parts, 10 parts *Rheum* sp. rhizome, and 10 parts *B. rotunda* (L.) Mansf. rhizome.

### 3.3. Preparation of PSKMHR Extracts

Seventy percent ethanol in water was employed as the extractive solvent, and the samples were subjected to extraction using a liquid–liquid extraction method. In this process, 5 g of each sample was combined with the extractive solvent at a ratio of 1:10 (*w*/*v*) and sonicated for 10 min. Following the extraction, the mixture was filtered with a vacuum pump to separate the solvent from the solid residue. The solid residue was then re-extracted three additional times. Subsequently, the combined solution was evaporated to dryness using a rotary evaporator. The resulting dry weight was measured and stored in a refrigerator at −20 degrees Celsius for the subsequent analysis.

### 3.4. HPLC-UV Analysis

The high-performance liquid chromatography (HPLC) system used in this study was an Agilent 1260 Infinity II series (Santa Clara, CA, USA), comprising a quaternary pump, an autosampler, a multi-column thermostat, and a photodiode array detector. The chromatographic separation was conducted using an ACE 5 C18-AR column (4.6 × 250 mm, 5 µm) sourced from Aberdeen, UK, coupled with a Phenomenex C18 guard column (4 mm × 3 mm × 5 µm) from Torrance, CA, USA. The mobile phase was composed of 0.1% formic acid in ultrapure water (phase A) and 0.1% formic acid in acetonitrile (phase B). The sample was pretreated and analyzed following specific gradient conditions: initially, the gradient was adjusted from 20% to 80% of phase B over a 30 min period, maintained at 80% phase B for 5 min, then returned to 20% phase B over the next 5 min, and held at 20% phase B for an additional 10 min. The analysis was conducted at a flow rate of 1 mL/min with a detection wavelength range of 200–600 nm, including specific wavelengths of 288, 326, 350, and 430 nm. The column temperature was maintained at 25 °C, and the injection volume was set at 10 µL.

### 3.5. Method Validation

The developed HPLC-PDA method was validated based on the AOAC guidelines, assessing parameters such as linearity, limit of detection (LOD), limit of quantification (LOQ), accuracy, and precision.

#### 3.5.1. Specificity

The specificity of the HPLC-PDA analysis was evaluated by comparing the results obtained from mixed standard solutions with corresponding sample solutions at specific wavelengths: quercitrin and wedelolactone at 350 nm; rhaponticin and rhapontigenin at 326 nm; alpinetin, pinocembrin, pinostrobin, and panduratin A at 288 nm; and aloe-emodin, rhein, emodin, chrysophanol, and physcion at 430 nm. The thirteen peaks of the standards were confirmed to be fully resolved from other peaks present in the chromatogram of the PSKMHR samples, based on their respective retention times and UV spectra. The specificity of both the standard and sample solutions was further validated through triplicate analyses.

#### 3.5.2. Linearity

Five-point calibration curves were constructed for various compounds in specified concentration ranges: rhein (0.05–0.50 μg/mL); aloe-emodin, emodin, and physcion (0.1–0.5 μg/mL); chrysophanol (0.25–1.25 μg/mL); apigenin (0.5–4.0 μg/mL); and quercitrin, rhapontigenin, wedelolactone, pinocembrin, and panduratin A (1.00–5.00 μg/mL). The concentration range was extended to 2.00–12.00 μg/mL for pinostrobin and 3.00–15.00 μg/mL for rhaponticin, with all measurements performed in triplicate.

#### 3.5.3. Limit of Detection (LOD) and Limit of Quantification (LOQ)

Utilizing the linear equation data, the limit of detection (LOD) and limit of quantification (LOQ) were calculated employing the following formulas:

LOD = 3.3 × (Standard Deviation of Y-intercept)/(Mean of Slope)

LOQ = 10 × (Standard Deviation of Y-intercept)/(Mean of Slope)

LODs and LOQs were calculated as 3.3 and 10 times the standard deviation of the calibration curve/mean slope of the calibration curve ratio, respectively.

#### 3.5.4. Accuracy

The recovery of the developed HPLC-PDA method was evaluated using spiked samples at three distinct concentration levels: 0.15, 0.30, and 0.45 μg/mL for aloe-emodin, rhein, emodin, and physcion; 0.4, 0.8, and 1.2 μg/mL for chrysophanol; 1.50, 3.00, and 4.50 μg/mL for quercitrin, rhapontigenin, wedelolactone, pinocembrin, and panduratin A; 1.00, 2.00, and 3.00 μg/mL for apigenin; 3.00, 7.00, and 11.00 μg/mL for pinostrobin; and 5.00, 9.00, and 13.00 μg/mL for rhaponticin. Recovery percentages were calculated by comparing the observed concentrations to the known concentrations of the spiked samples. The accuracy of the method was affirmed by conducting triplicate analyses.

#### 3.5.5. Precision

The precision of the method was evaluated as repeatability through triplicate injections conducted consecutively within a single day, and as intermediate precision by performing triplicate injections across three different days at low, medium, and high concentration levels. The relative standard deviation (RSD%) values were calculated to assess precision. The accuracy of the method was determined through the mean recovery percentage (% recovery), which was established by conducting triplicate injections at these low, medium, and high concentration levels.

### 3.6. Statistical Analysis

Data were expressed as the mean, SD, and RSD (%), using Microsoft Excel 365 software (Microsoft Co., Redmond, WA, USA).

## 4. Conclusions

The ethanolic extract of PSKMHR was analyzed using high-performance liquid chromatography with photodiode array detection (HPLC-PDA), which led to the identification of several compounds, including quercitrin from *C. quadrangularis*, wedelolactone from *E. prostrata*, two stilbenes (rhaponticin and rhapontigenin), and five anthraquinones (aloe-emodin, rhein, emodin, chrysophanol, and physcion) derived from *Rheum* spp. Furthermore, alpinetin, pinocembrin, pinostrobin, and panduratin A were also identified from *B. rotunda*. The compounds exhibited specific maximum absorbances: quercitrin and wedelolactone at approximately 350 nm; rhaponticin and rhapontigenin at approximately 326 nm; aloe-emodin, rhein, emodin, chrysophanol, and physcion at approximately 430 nm; and alpinetin, pinocembrin, pinostrobin, and panduratin A at approximately 288 nm. The analytical method, validated in accordance with AOAC standards, demonstrated strong specificity, linearity, sensitivity, accuracy, and precision, confirming its suitability for quality control of PSKMHR samples. Based on the chromatographic data and the quantified concentrations of the thirteen markers, the PSKMHR samples were categorized into two distinct groups. The samples with higher stilbene concentrations were designated as Group 1, while those with lower stilbene concentrations were classified as Group 2, potentially attributable to the variation in rhubarb species used. These findings underscore the necessity for the standardization of raw materials in manufacturing processes to ensure consistent quality and efficacy across commercial herbal remedies.

## Figures and Tables

**Table 1:** The retention time, detection wavelength, linear range, regression equation, coefficient of determination (R^2^), limit of detection (LOD), and limit of quantification (LOQ) for the simultaneous analysis of thirteen authentic standards by HPLC (*n* = 3).

Retention Time (min)	Analyte	Detection Wavelength (nm)	Range (µg/mL)	Linear Equation	R^2^	LOD (µg/mL)	LOQ (µg/mL)
8.17	Rhaponticin (**1**)	326	3.00–15.00	Y = 169.49X − 1.4532	1.0000	0.0113	0.0342
				Y = 170.16X − 1.4590			
				Y = 168.81X − 1.4474			
				Mean slope = 103.26			
				SD intercept = 0.0058			
8.56	Quercitrin (**2**)	350	1.00–5.00	Y = 103.16X − 0.9693	0.9999	0.0113	0.0341
				Y = 103.16X − 0.9763			
				Y = 103.46X − 0.9722			
				Mean slope = 103.26			
				SD intercept = 0.0035			
12.99	Rhapontigenin (**3**)	326	1.00–5.00	Y = 149.13X − 0.1382	0.9999	0.0090	0.0270
				Y = 149.87X − 0.1389			
				Y = 149.84X − 0.1389			
				Mean slope = 149.62			
				SD intercept = 0.0004			
13.71	Wedelolactone (**4**)	350	1.00–5.00	Y = 100.29X − 0.6422	1.0000	0.0086	0.0259
				Y = 99.87X − 0.6396			
				Y = 100.69X − 0.6448			
				Mean slope = 100.29			
				SD intercept = 0.0026			
15.69	Alpinetin (**5**)	288	0.50–4.00	Y = 212.52X − 1.8802	0.9999	0.0134	0.0405
				Y = 213.89X − 1.8915			
				Y = 211.98X − 1.8746			
				Mean slope = 212.80			
				SD intercept = 0.0086			
20.17	Aloe-emodin (**6**)	430	0.10–0.50	Y = 142.30X − 0.5479	0.9999	0.0039	0.0118
				Y = 142.87X − 0.5501			
				Y = 143.15X − 0.5512			
				Mean slope = 142.77			
				SD intercept = 0.0017			
20.86	Rhein (**7**)	430	0.05–0.50	Y = 150.65X − 0.0548	0.9999	0.0039	0.0118
				Y = 149.90X − 0.0545			
				Y = 150.05X − 0.0545			
				Mean slope = 150.20			
				SD intercept = 0.0002			
21.51	Pinocembrin (**8**)	288	1.00–5.00	Y = 207.87X − 0.0934	0.9999	0.0073	0.0221
				Y = 206.83X − 0.0929			
				Y = 208.70X − 0.0938			
				Mean slope = 207.80			
				SD intercept = 0.0005			
26.93	Emodin (**9**)	430	0.10–0.50	Y = 142.63X − 0.1773	0.9999	0.0032	0.0100
				Y = 143.34X − 0.1782			
				Y = 142.20X − 0.1768			
				Mean slope = 142.72			
				SD intercept = 0.0007			
27.79	Pinostrobin (**10**)	288	2.00–12.00	Y = 204.13X − 3.4118	1.0000	0.0125	0.0377
				Y = 203.31X − 3.3982			
				Y = 205.15X − 3.4289			
				Mean slope = 204.20			
				SD intercept = 0.0154			
31.73	Chrysophanol (**11**)	430	0.25–1.25	Y = 150.28X − 0.1692	0.9999	0.0091	0.0269
				Y = 150.73X − 0.1697			
				Y = 151.03X − 0.1700			
				Mean slope = 150.68			
				SD intercept = 0.0004			
33.00	Panduratin A (**12**)	288	1.00–5.00	Y = 193.98X − 0.3301	0.9999	0.0040	0.0118
				Y = 193.40X − 0.3290			
				Y = 194.75X − 0.3301			
				Mean slope = 194.04			
				SD intercept = 0.0012			
34.37	Physcion (**13**)	430	0.10–0.50	Y = 136.61X − 0.0074	0.9999	0.0060	0.0180
				Y = 135.93X − 0.0071			
				Y = 136.61X − 0.0069			
				Mean slope = 136.66			
				SD intercept = 0.0003			

**Table 2:** Accuracy and precision for marker analytes in PSKMHR (*n* = 3).

Analyte	Spiked (µg/mL)	Day	Found (µg/mL)	SD	Intra-Day %RSD	%Recovery	Inter-Day %RSD
**1**	5.00	1	4.98	0.03	0.69	99.63	0.92
		2	4.94	0.06	1.26	98.91	
		3	5.00	0.04	0.73	99.92	
	9.00	1	8.95	0.07	0.80	99.44	0.78
		2	9.00	0.02	0.27	100.07	
		3	8.95	0.10	1.17	99.48	
	13.00	1	12.94	0.06	0.46	99.52	0.44
		2	12.94	0.06	0.50	99.53	
		3	12.92	0.07	0.53	99.37	
**2**	1.50	1	1.48	0.03	1.84	98.82	1.11
		2	1.50	0.01	0.55	99.67	
		3	1.50	0.01	0.62	99.82	
	3.00	1	2.98	0.04	1.17	99.87	1.43
		2	3.03	0.04	1.45	101.06	
		3	2.97	0.04	1.18	99.09	
	4.50	1	4.47	0.02	0.44	99.34	0.38
		2	4.48	0.01	0.32	99.62	
		3	4.49	0.02	0.38	99.75	
**3**	1.50	1	1.48	0.01	0.85	98.84	0.74
		2	1.48	0.01	0.50	98.91	
		3	1.48	0.01	0.58	99.80	
	3.00	1	2.97	0.01	0.48	98.99	0.86
		2	2.97	0.02	0.59	99.01	
		3	3.01	0.02	0.64	100.40	
	4.50	1	4.48	0.01	0.31	99.60	0.29
		2	4.49	0.01	0.27	99.71	
		3	4.50	0.02	0.32	99.89	
**4**	1.50	1	1.52	0.02	1.55	101.16	1.24
		2	1.50	0.02	1.30	99.89	
		3	1.50	0.01	0.83	100.07	
	3.00	1	3.02	0.03	1.01	100.67	1.26
		2	3.00	0.02	0.70	100.14	
		3	2.96	0.04	1.43	98.79	
	4.50	1	4.52	0.02	0.48	100.39	0.38
		2	4.51	0.01	0.32	100.21	
		3	4.50	0.02	0.34	99.99	
**5**	1.00	1	1.00	0.02	1.64	100.77	1.19
		2	1.01	0.01	1.16	100.93	
		3	1.01	0.01	1.27	100.87	
	2.00	1	2.02	0.01	0.52	101.15	0.53
		2	2.01	0.01	0.46	100.55	
		3	2.02	0.01	0.52	101.15	
	3.00	1	3.00	0.01	0.37	100.09	0.49
		2	2.99	0.02	0.68	99.76	
		3	3.00	0.01	0.49	100.13	
**6**	0.15	1	0.15	0.00	1.99	100.67	1.65
		2	0.15	0.00	1.75	100.67	
		3	0.15	0.00	1.92	100.22	
	0.30	1	0.30	0.00	1.00	100.33	1.31
		2	0.30	0.01	1.66	100.33	
		3	0.30	0.00	1.58	99.56	
	0.45	1	0.45	0.01	1.26	100.30	1.05
		2	0.45	0.01	1.12	100.30	
		3	0.45	0.01	1.23	100.44	
**7**	0.15	1	0.15	0.00	1.67	100.44	1.55
		2	0.15	0.00	2.01	101.11	
		3	0.15	0.00	1.31	102.00	
	0.30	1	0.30	0.00	0.57	100.67	1.11
		2	0.30	0.00	0.83	101.11	
		3	0.30	0.01	1.83	100.22	
	0.45	1	0.45	0.00	0.90	100.07	0.85
		2	0.45	0.00	0.80	100.67	
		3	0.46	0.00	0.79	101.11	
**8**	1.50	1	1.50	0.02	1.17	99.98	0.91
		2	1.50	0.01	0.87	100.00	
		3	1.49	0.01	0.81	99.29	
	3.00	1	2.98	0.03	1.15	99.47	1.00
		2	2.99	0.03	0.86	99.60	
		3	3.00	0.04	1.31	99.98	
	4.50	1	4.47	0.03	0.68	99.35	0.55
		2	4.47	0.02	0.52	99.41	
		3	4.48	0.03	0.67	99.49	
**9**	0.15	1	0.15	0.00	1.38	100.22	1.49
		2	0.15	0.00	1.75	100.67	
		3	0.15	0.00	1.92	100.22	
	0.30	1	0.30	0.00	0.50	101.11	0.66
		2	0.30	0.00	1.00	101.44	
		3	0.30	0.00	0.57	101.22	
	0.45	1	0.46	0.00	0.88	101.11	0.63
		2	0.45	0.00	0.55	100.96	
		3	0.46	0.00	0.67	101.19	
**10**	3.00	1	2.99	0.02	0.76	99.67	0.72
		2	2.98	0.03	0.86	99.41	
		3	2.98	0.02	0.77	99.24	
	7.00	1	7.00	0.02	0.30	100.06	0.29
		2	6.98	0.02	0.27	99.73	
		3	6.98	0.02	0.26	99.77	
	11.00	1	10.98	0.05	0.46	99.81	0.31
		2	10.98	0.04	0.35	99.80	
		3	11.00	0.01	0.13	100.01	
**11**	0.40	1	0.40	0.01	1.25	100.83	1.14
		2	0.40	0.01	1.27	100.67	
		3	0.40	0.01	1.37	100.42	
	0.80	1	0.80	0.01	1.00	100.42	0.77
		2	0.80	0.01	0.93	100.46	
		3	0.81	0.01	0.62	100.75	
	1.20	1	1.20	0.01	1.23	100.17	0.68
		2	1.20	0.00	0.29	100.36	
		3	1.21	0.00	0.29	100.61	
**12**	1.50	1	1.49	0.02	1.48	99.51	1.26
		2	1.49	0.02	1.37	99.42	
		3	1.49	0.02	1.58	99.62	
	3.00	1	2.99	0.03	1.14	99.77	0.82
		2	2.99	0.01	0.47	99.67	
		3	3.01	0.03	0.91	100.27	
	4.50	1	4.48	0.01	0.30	99.64	0.38
		2	4.48	0.02	0.41	99.64	
		3	4.48	0.03	0.56	99.63	
**13**	0.15	1	0.15	0.00	1.38	100.22	1.24
		2	0.15	0.00	1.32	100.67	
		3	0.15	0.00	1.53	100.44	
	0.30	1	0.30	0.00	0.50	101.11	1.14
		2	0.30	0.00	1.65	99.89	
		3	0.30	0.00	1.07	100.56	
	0.45	1	0.46	0.00	0.67	101.04	0.86
		2	0.45	0.00	0.84	100.52	
		3	0.45	0.00	1.02	100.00	

**Table 3:** Concentrations of 13 analytes in the crude extracts of PSKMHR formulated from ten market raw material samples at 10 mg/mL (reported as µg ± SD per g of dried weight, *n* = 3).

Analyte	S–1	S–2	S–3	S–4	S–5	S–6	S–7	S–9	S–9	S–10
Rhaponticin (**1**)	890.34 ± 17.75	892.12 ± 17.78	<LOQ	863.60 ± 17.21	845.78 ± 16.86	<LOQ	<LOQ	952.72 ± 18.99	925.98± 18.46	881.43 ± 17.57
Quercitrin (**2**)	183.41 ± 2.44	183.97 ± 2.44	184.25 ± 2.44	174.96 ± 2.44	169.33 ± 2.44	172.14 ± 2.44	191.85 ± 2.45	203.11 ± 2.46	194.67 ± 2.45	180.59 ± 2.44
Rhapontigenin (**3**)	301.39 ± 4.31	301.99 ± 4.32	<LOQ	292.34 ± 4.18	286.31 ± 4.10	<LOQ	<LOQ	322.49 ± 4.61	313.45 ± 4.48	298.37 ± 4.27
Wedelolactone (**4**)	251.41 ± 5.03	251.92 ± 5.04	252.17 ± 5.04	243.85 ± 4.88	238.81 ± 4.78	241.33 ± 4.83	258.98 ± 5.18	269.06 ± 5.38	261.50 ± 5.23	248.89 ± 4.98
Alpinetin (**5**)	164.92 ± 3.83	165.25 ± 3.84	165.42 ± 3.84	159.95 ± 3.71	156.63 ± 3.64	158.29 ± 3.68	169.89 ± 3.94	176.53 ± 4.10	171.55 ± 3.98	163.26 ± 3.79
Aloe-emodin (**6**)	20.76 ± 0.32	20.81 ± 0.32	20.83 ± 0.32	20.13 ± 0.31	19.71 ± 0.30	19.92 ± 0.30	21.40 ± 0.33	22.24 ± 0.34	21.61 ± 0.33	20.55 ± 0.31
Rhein (**7**)	2.86 ± 0.04	2.86 ± 0.04	26.09 ± 0.34	2.77 ± 0.04	2.71 ± 0.03	24.83 ± 0.33	26.99 ± 0.35	3.06 ± 0.04	2.97 ± 0.04	2.83 ± 0.04
Pinocembrin (**8**)	392.44 ± 8.85	393.23 ± 8.87	393.62 ± 8.88	380.67 ± 8.59	372.82 ± 8.41	376.74 ± 8.50	404.22 ± 9.12	419.92 ± 9.47	408.14 ± 9.21	388.52 ± 8.76
Emodin (**9**)	28.29 ± 0.57	28.35 ± 0.57	42.96 ± 0.87	27.44 ± 0.56	26.87 ± 0.54	41.11 ± 0.83	44.12 ± 0.89	30.28 ± 0.61	29.43 ± 0.60	28.01 ± 0.57
Pinostrobin (**10**)	890.55 ± 20.13	892.33 ± 20.17	893.22 ± 20.19	863.78 ± 19.53	845.94 ± 19.12	854.86 ± 19.32	917.31 ± 20.73	953.00 ± 21.54	926.23 ± 20.93	881.62 ± 19.93
Chrysophanol (**11**)	94.03 ± 1.18	94.22 ± 1.18	50.44 ± 0.63	91.21 ± 1.14	89.33 ± 1.12	61.11 ± 0.77	68.63 ± 0.86	100.62 ± 1.26	97.80 ± 1.23	93.09 ± 1.17
Panduratin A (**12**)	287.05 ± 5.01	287.62 ± 5.02	287.91 ± 5.02	278.43 ± 4.86	272.69 ± 4.76	275.56 ± 4.81	295.67 ± 5.16	307.15 ± 5.36	298.54 ± 5.21	284.18 ± 4.96
Physcion (**13**)	28.25 ± 0.78	28.31 ± 0.78	18.20 ± 0.50	27.40 ± 0.75	26.84 ± 0.74	17.42 ± 0.48	18.69 ± 0.51	30.23 ± 0.83	29.38 ± 0.81	27.97 ± 0.77

## Data Availability

Data are contained within the article.
